# Fungal keratitis due to *Scopulariopsis brevicaulis* and a potential promising therapeutic effect of antibacterial agents: A case report

**DOI:** 10.1097/MD.0000000000028203

**Published:** 2021-12-10

**Authors:** Nasser M. Kaplan, Rami A. Al-Dwairi, Nasr N. AlRabadi

**Affiliations:** a Department of Pathology and Microbiology, King Abdullah University Hospital, Jordan University of Science and Technology, Irbid, Jordan; b Department of Special Surgery (Ophthalmology), King Abdullah University Hospital, Jordan University of Science and Technology, Irbid, Jordan; c Department of Pharmacology, Faculty of Medicine, Jordan University of Science and Technology, Irbid, Jordan.

**Keywords:** contact lens, keratitis, *Scopulariopsis brevicaulis*

## Abstract

**Introduction:**

Microbial keratitis is a serious potentially blinding corneal infection. Contact lens wear remains the most common predisposing factor. Fungal keratitis represent only a small fraction of the overall number of cases of contact lens-associated microbial keratitis, however they are proportionally more severe.

**Patient concerns:**

An 18-year-old female, who occasionally used eye cosmetic soft contact lenses, presented with pain, redness, and blurring of vision in her left eye.

**Diagnosis:**

The left eye showed decreased visual acuity, central corneal ulcer and abscess, and severe ciliary injection. A provisional diagnosis of infectious keratitis was considered.

**Intervention:**

Corneal scrapings were aseptically collected and directly inoculated onto sterile bacterial and fungal agar plates that were immediately incubated. The patient was admitted and started on topical and systemic antibacterial agents.

**Outcomes:**

The infection showed signs of satisfactory clinical resolution. However, the mold *Scopulariopsis brevicaulis* was isolated in pure colonies 5 days after presentation.

**Conclusion:**

We report the first case from Jordan of fungal keratitis caused by the mold *S brevicaulis*. A high index of suspicion is required for fungal keratitis caused by *S brevicaulis* in immunocompetent patients who wear contact lenses despite its rarity. This fungal infection was successfully treated using antibacterial agents. However, larger studies are recommended to investigate the clinical effectiveness of antimicrobial agents that have both antibacterial and antifungal effects and to assess their role as empirical therapeutic modalities for infectious keratitis.

## 1 Introduction

Microbial keratitis is a serious and potentially blinding corneal infection. Predisposing factors include ocular surface disease, ocular trauma, contact lens wear, systemic diseases, and ocular surgery.^[1]^ Although it represents an easily preventable cause of microbial keratitis, contact lens wear remains the most common predisposing factor of this infection along with ocular trauma.^[2,3]^ Indeed, wearing contact lens overnight and poor hygiene are the 2 most common risk factors of contact lens-associated microbial keratitis (CLMK).

Statistics show that around 90% of CLMK is due to bacteria, however the facultative protozoa *Acanthamoebae* and fungi represent only a small fraction of the overall number of cases of CLMK but are proportionally more severe.^[4]^ Infection with *Aspergillus* and *Candida* species remains the most common cause of fungal CLMK, however, infection with other fungal species is possible and was previously reported. For example, the species of *Scopulariopsis,* is an uncommon cause of invasive disseminated infections but are still recognized as relatively common opportunistic causes of keratitis, onychomycosis, and otomycosis.^[5–9]^

*Scopulariopsis brevicaulis* is the most prevalent and significant clinical species isolated from superficial and invasive infections with this saprophytic mold.^[9,10]^ Previous reports of fungal keratitis caused by *S brevicaulis* indicate that it is an opportunistic pathogen that will not normally infect a healthy cornea and that fungal inoculation most likely follows corneal trauma. Among the cases of *S brevicaulis* keratitis reported in the literature; one followed a penetrating eye injury with a dirty rusty nail^[11]^ and another one followed corneal damage caused by herpes virus type I.^[12]^

*S brevicaulis* is a cosmopolitan saprophytic mold that is ubiquitous in the environment and commonly found in soil. In view of the relatively low incidence of keratitis caused by *S brevicaulis* and the high frequency of multiple drug resistance in species isolated from other infection sites, there is little consensus on the best therapeutic modality. In general terms, fungal keratitis is initially treated with antifungal agents that include itraconazole, miconazole, chlorhexidine, sulphadiazine, econazole, or natamycin for several months. Patients who do not improve following treatment with topical and/or oral antifungal medications may require surgery, including corneal transplantation.^[13]^ Recently, many clinical cases reported the responsiveness of fungal keratitis for antibacterial treatment.^[14–21]^

Sporadic ocular infections have been previously reported,^[11,12,22]^ however it is not uncommon to have a delay in the diagnosis of fungal keratitis because of occasional difficulty in collecting the corneal samples as well as isolating and identifying the pathogenic fungus.

## 2 Case report and microbiology findings

An 18-year-old female presented to the ophthalmology outpatient clinic with a 2-day history of pain, redness, and blurring of vision in her left eye. The patient had no significant past medical history and was an occasional cosmetic soft contact lenses wearer. The patient used to live in a rural area, had long unclean fingers’ nails, and worn contact lenses on the same day the symptoms had started. On eye examination, the visual acuity was 1.0 in the right eye and 0.1 in the left eye. The left eye showed a central 3 × 3 mm corneal ulcer and a 1 × 1 mm underlying corneal abscess with generalized corneal edema and a 1-mm hypopyon level in the anterior chamber. There was also associated mild eyelid edema and severe ciliary injection.

A provisional diagnosis of infectious keratitis was considered. Under strict aseptic conditions, corneal scrapings were collected and inoculated directly onto sterile agar plates of bacterial and fungal culture media. The inoculated plates were promptly transported and delivered to the clinical microbiology laboratory (CML) for immediate incubation. The patient was admitted for therapy as well as close and frequent observations.

The therapeutic regimen included antimicrobial agents that were administered topically as hourly eye drops of fortified amikacin (aminoglycoside) and fortified vancomycin (glycopeptide), and systemically as 750 mg twice-daily oral ciprofloxacin (fluoroquinolone). Cyclopentolate hydrochloride 1% was also administered topically as hourly eye drops for relief of pain. On the next day, the patient was symptomatically improving. The corneal ulcer and abscess were the same size but the hypopyon was almost resolved. On the second day, the visual acuity had improved to 0.4, the ulcer was almost 2 × 2 mm and the abscess was 0.5 × 0.5 mm with the hypopyon completely disappeared. Corneal thinning at the site of the ulcer was noticed and intensive lubricating eye drops were added to the therapeutic regimen. The patient was discharged on the third day of the same treatment course.

Two days later, the patient was presented with a mild deterioration of vision. Despite the signs of clinical improvement, corneal melting was noticed at the site of the almost healed ulcer. All fortified eye drops were discontinued and replaced by moxifloxacin (fluoroquinolone) eye drops 4 times daily. Lubricating eye drops were continued hourly.

On further follow-up, the corneal defect had completely healed but thinning due to melting was obvious. Mild opacification of the cornea at the site of the previous ulcer had started to form. All eye drops other than lubricating agents were discontinued and the patient was stable in the follow-up visits.

The inoculated bacterial agar plates of Blood and MacConkey culture media (Oxoid, UK) were sterile and showed no bacterial growth after 48 hours of aerobic incubation at 34 to 36°C. The inoculated bacterial agar plates of Chocolate culture media (Oxoid, UK) were also sterile and showed no bacterial growth after 48 hours of incubation under 5% CO_2_ at 34 to 36°C.

**Figure 1:**
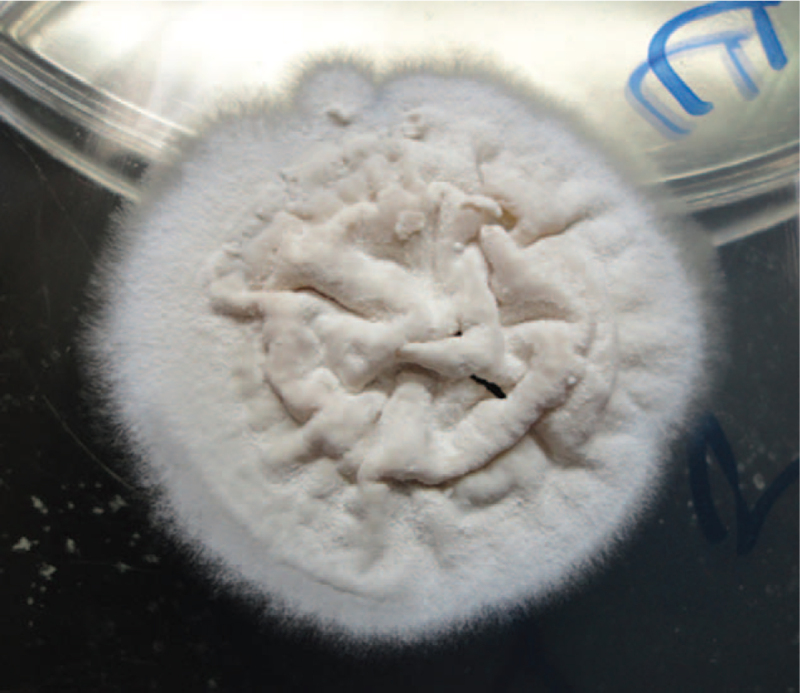
Off-white colony with powdery surface and feathery borders on Sabouraud dextrose agar plate.

**Figure 2:**
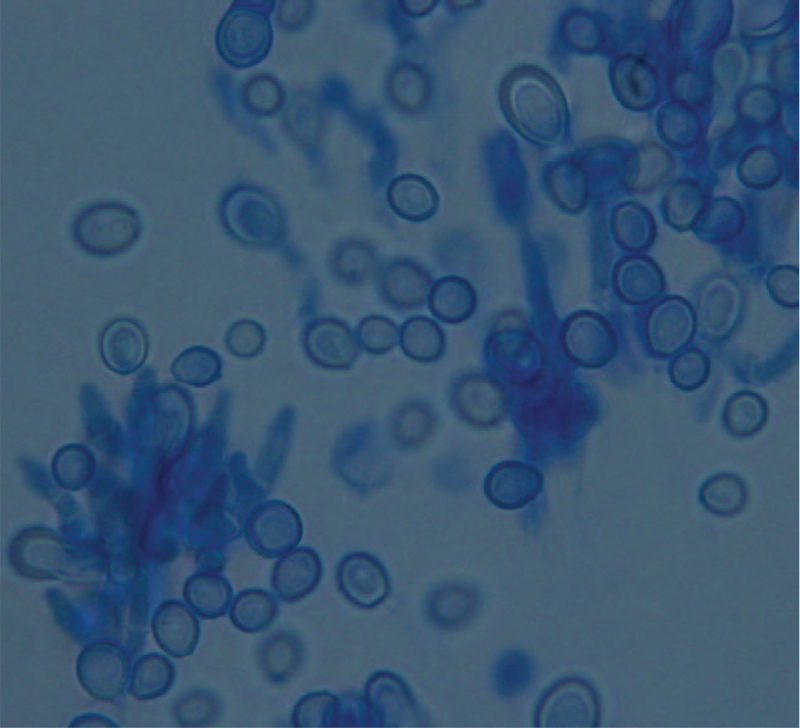
Branched conidiophores and chains of characteristic conidia, lactophenol-cotton blue stain, ×400 magnification.

However after 5 days, identical fungal growths were detected on the inoculated fungal agar plates of Sabouraud Dextrose culture media (Oxoid, UK) incubated under aerobic conditions at 25°C, as well as on the other blood and chocolate agars kept on the laboratory bench. The isolated moderately fast-growing mold was identified as *S brevicaulis* based on both the macroscopic characteristic colony morphology and the microscopic observation of distinctive fungal elements. The colonies were off-white with powdery surface and feathery borders (Fig. 1). Light microscopic examination of the colonies stained with lactophenol cotton blue showed hyaline septate hyphae, numerous branched conidiophores, and chains of rough thick-walled lemon-shaped conidia with flattened bases and rounded or slightly pointed tips (Fig. 2).

The contact lens storing fluid was received and centrifuged, and the resulting sediment was cultured. Dark-colored soil debris from underneath the distal edges of the long fingers’ nails of the patient was also collected and cultured. The storing fluid's sediment and soil debris were inoculated onto separate Sabouraud dextrose agar plates, and the same etiologic mold *S brevicaulis* was isolated after 5 days of aerobic incubation at 25°C. The storing fluid's sediment and soil debris were also inoculated onto separate non-nutrient agar plates (Oxoid, UK) overlaid with Escherichia coli, and the plates were negative for trophozoites and cysts of the facultative protozoa *Acanthamoebae*.

Ethical approval by the institutional review board was not required because this is a case report. However a written informed consent was obtained from the patient to participate in this study.

## 3 Discussion

This reported case was diagnosed as a confirmed contact lens-associated fungal keratitis caused by *S brevicaulis*, according to the criteria listed for “proven fungal disease” caused by molds,^[23]^ because this fungus was the only pathogen recovered by the culture of corneal scrapings specimen that was obtained by a sterile procedure from clinically abnormal site consistent with an infectious disease process. The same fungus was also isolated from both the patient nails’ soil debris and the contact lens storing fluid, therefore the nails’ soil debris was considered the most likely environmental source of the fungus that was subsequently transmitted to the contact lens storing fluid. The sterility of bacterial culture media indicates the absence of any other probable conventional bacterial pathogens. Negative special cultures also excluded the possibility of *Acanthamoebae* infection.

An important limitation in this case was that the CML did not receive glass slides smeared with the aseptically collected corneal scrapings for preliminary diagnostic staining techniques. Therefore, there were no available results of the direct microscopic examination for bacterial organisms or fungal filaments. However in cases of fungal infections, direct microscopic examination of the tissue generally detect nonspecific fungal elements and usually will not distinguish between various molds. Therefore, modern molecular sequencing methods were previously reported to be useful for rapid and accurate identification of *Scopulariopsis* species directly from tissue and from culture.^[24]^ Another limitation in this case was that antimicrobial susceptibility testing of the isolated mold was not performed in our diagnostic CML because the facilities required are available only in a reference Mycology laboratory.

CLMK requires urgent treatment to contain damage and to improve prognosis, however it usually poses a clinical diagnostic dilemma as the distinction between sterile inflammation and microbial infection is often unclear.^[4,25]^ Initial treatment of suspected cases of microbial keratitis with topical fortified antimicrobial agents has long been the gold standard. In this case, antibacterial treatment was commenced empirically as bacterial keratitis is the most common form of infectious keratitis and because it is considered an ocular emergency due to its rapid progression and disastrous complications.^[13]^ The guidelines on sterile preparation of fortified antimicrobial eye drops to achieve the adequate higher drug concentration for topical therapy have been recently reported.^[26]^

Fungal keratitis is often highly invasive and antifungal agents tend to be fungistatic which can lead to prolonged treatment and often surgical intervention.^[27]^ Natamycin, 5% polyene suspension, is most commonly the first choice of treatment of filamentous fungal keratitis.^[28]^ Successful treatment of *S brevicaulis* keratitis by topical natamycin was reported,^[22]^ however, natamycin is not available in Jordan. Amphotericin B is less effective against filamentous fungi; however, it is particularly effective and is the first agent of choice against yeasts.^[29]^ The treatment of *Scopulariopsis* infection is challenging and is not well understood because of its rarity and the high level of resistance of *Scopulariopsis* species to conventional antifungal agents.^[9,30,31]^ Antifungal therapy alone rarely achieves a cure and other therapeutic interventions, as debridement or excision of necrotic tissue, are also required. In fungal keratitis, the surgical debridement of corneal ulcer helps to significantly reduce the load of infecting fungus and enhance the penetration of topical therapeutic agents.^[32]^

The infection was clinically resolved in this patient due to early presentation, prompt diagnosis, and immediate empirical treatment with topical antimicrobial eye drops. There have been anecdotal reports of fungal ulcers that improved with antibiotic treatment alone.^[33]^ However, further in-vitro and clinical studies are still required for the standardization of the use of these antimicrobial agents in the effective therapeutic modalities of fungal keratitis.

Fungi are susceptible to the traditional aminoglycosides at high concentrations,^[34]^ and the antifungal activities of aminoglycosides have been recently reported.^[35,36]^ Forster et al^[19]^ reported 2 patients with *Curvularia pallescens* and *Drechslera halodes* keratitis cured with topical gentamicin therapy. Chodosh et al^[15]^ reported a cure of *Fusarium* species keratitis with topical tobramycin and vancomycin. Munir et al^[17]^ reported 5 cases of fungal keratitis responsive to antibiotic therapy. Of the 2 cases that were corneal culture positive, 1 case of *Fusarium* keratitis was cured by tobramycin and cefazolin. In 2012, Lee et al^[21]^ reported another case of *Fusarium* keratitis cured with moxifloxacin monotherapy. Motaba^[14]^ reported cures of *Curvularia* species, *Candida parapsilosis*, *Aspergillus*, and *Paecilomyc lilacinus* keratitis with moxifloxacin monotherapy. Khor et al^[16]^ described 68 eyes of 66 patients with *Fusarium* keratitis. In 11 eyes (16.2%), topical antibiotic therapy with fortified cefazolin and gentamicin led to the resolution of the infection. Rao et al^[18]^ described 12 patients with *Fusarium* keratitis, 4 of whom (33%) were cured with a combination of antibiotics consisting of a fluoroquinolone agent (levofloxacin or ofloxacin), tobramycin, and vancomycin. Choy et al^[20]^ reported a cure for *Fusarium* keratitis in 5 of 14 patients (36%) treated with antibiotics by typically tobramycin and ceftazidime.

The fluoroquinolones are irreversibly-bactericidal antimicrobial agents that can bind to both prokaryotic and eukaryotic topoisomerases and may have some antifungal activity. Current commercial topical preparations of fluoroquinolones demonstrated a definite antifungal activity against *Fusarium* and *Candida* isolates from human ocular infections.^[37,38]^ The in-vitro activity of tobramycin and moxifloxacin against filamentous fungi was also reported.^[33]^ The inhibitory effects of fluoroquinolones and aminoglycosides against the *Fusarium* species isolated from eyes with fungal keratitis were reported, however at relatively high concentrations.^[39]^

In this case, the patient was continued on antibacterial therapy because of the noticed satisfactory clinical response, the relatively delayed fungal growth, and to avoid the well-known toxic effects of antifungal agents particularly associated with the required long therapeutic duration. Amphotericin B, for example, has significant toxic effects that include dose-limiting nephrotoxicity, punctate epithelial erosions, and a greenish discoloration of the cornea.^[13]^

Generally, effective preventive measures remain the cornerstone in combating CLMK. Full recommendations regarding the proper usage of contact lenses for the prevention of potential infections have been studied and reviewed recently.^[2]^ Consequently, our patient was advised regarding the specific attention to contact lens hygiene including both storage case cleaning and replacement for daily lens use, avoidance of overnight lens wearing, consideration of using daily disposable lenses, and other hand hygiene measures including the short trimming and frequent cleaning of fingers’ nails.

## 4 Conclusion

Infectious keratitis is one of the major causes of avoidable blindness. Delay in clinical and microbiology diagnosis is responsible for inappropriate initial therapy and undesirable outcome. The significance of liaison between the ophthalmologists and microbiologists for early isolation and proper identification of fungal etiology of corneal infection should be highly emphasized. The role of antimicrobial agents that have both antibacterial and antifungal effects, as empirical therapeutic modalities for infectious keratitis should be further studied. The contact lens wearers should pay attention to storage case hygiene practices and avoid delays in presentation for early appropriate treatment.

## Author contributions

**Conceptualization:** Nasser M. Kaplan, Rami A. Al-Dwairi, Nasr N. AlRabadi

**Data curation:** Nasser M. Kaplan, Rami A. Al-Dwairi, Nasr N. AlRabadi

**Formal analysis:** Nasser M. Kaplan, Rami A. Al-Dwairi, Nasr N. AlRabadi

**Investigation:** Nasser M. Kaplan, Rami A. Al-Dwairi, Nasr N. AlRabadi

**Methodology:** Nasser M. Kaplan, Rami A. Al-Dwairi, Nasr N. AlRabadi

**Project Administration:** Nasser M. Kaplan, Rami A. Al-Dwairi, Nasr N. AlRabadi

**Resources:** Nasser M. Kaplan, Rami A. Al-Dwairi, Nasr N. AlRabadi

**Software:** Nasser M. Kaplan, Rami A. Al-Dwairi, Nasr N. AlRabadi

**Supervision:** Nasser M. Kaplan, Rami A. Al-Dwairi, Nasr N. AlRabadi

**Validation:** Nasser M. Kaplan, Rami A. Al-Dwairi, Nasr N. AlRabadi

**Visualization:** Nasser M. Kaplan, Rami A. Al-Dwairi, Nasr N. AlRabadi

**Writing – original draft:** Nasser M. Kaplan, Rami A. Al-Dwairi, Nasr N. AlRabadi

**Writing – review & editing:** Nasser M. Kaplan, Rami A. Al-Dwairi, Nasr N. AlRabadi

## References

[1] Stapleton F Carnt N. Contact lens-related microbial keratitis: how have epidemiology and genetics helped us with pathogenesis and prophylaxis? Eye 2012;26:185–93. 22134592 10.1038/eye.2011.288 PMC3272197

[2] Lievens CW Cilimberg KC Moore A. Contact lens care tips for patients: an optometrist's perspective. Clin Optom 2017;9:113–21. 10.2147/OPTO.S139651 PMC6118862 30214367

[3] Keay L Stapleton F. Development and evaluation of evidence-based guidelines on contact lens-related microbial keratitis. Contact Lens Anterior Eye 2008;31:03–12. 10.1016/j.clae.2007.10.003 18032091

[4] Carnt N Samarawickrama C White A. The diagnosis and management of contact lens-related microbial keratitis. Clin Exp Optom 2017;100:482–93. 28815736 10.1111/cxo.12581

[5] Cuenca-Estrella M Gomez-Lopez A Buitrago MJ. In vitro activities of 10 combinations of antifungal agents against the multiresistant pathogen *Scopulariopsis brevicaulis*. Antimicrob Agents Chemother 2006;50:2248–50. 16723597 10.1128/AAC.00162-06 PMC1479145

[6] Bunya VY Hammersmith KM Rapuano CJ. Topical and oral voriconazole in the treatment of fungal keratitis. Am J Ophthalmol 2007;143:151–3. 17188052 10.1016/j.ajo.2006.07.033

[7] Issakainen J Salonen JH Anttilas V-J. Deep, respiratory tract and ear infections caused by Pseudallescheria (*Scedosporium*) and *Microascus* (*Scopulariopsis*) in Finland. A 10-year retrospective multi-center study. Med Mycol 2010;48:458–65. 19672782 10.1080/13693780903161208

[8] Steinbach WJ Schell WA Miller JL. Fatal *Scopulariopsis brevicaulis* infection in a paediatric stem-cell transplant patient treated with voriconazole and caspofungin and a review of *Scopulariopsis* infections in immunocompromised patients. J Infect 2004;48:112–6. 14667801 10.1016/s0163-4453(03)00134-8

[9] Sandoval-Denis M Sutton DA Fothergill AW. *Scopulariopsis*, a poorly known opportunistic fungus: spectrum of species in clinical samples and in vitro response to antifungal drugs. J Clin Microbiol 2013;51:3937–43. 24025910 10.1128/JCM.01927-13 PMC3838093

[10] Iwen PC Schutte SD Florescu DF. Invasive *Scopulariopsis brevicaulis* infection in an immunocompromised patient and review of prior cases caused by *Scopulariopsis* and *Microascus* species. Med Mycol 2012;50:561–9. 22524638 10.3109/13693786.2012.675629

[11] Ragge NK Hart JCD Easty DL. A case of fungal keratitis caused by *Scopulariopsis brevicaulis*: treatment with antifungal agents and penetrating keratoplasty. Br J Ophthalmol 1990;74:561–2. 2168203 10.1136/bjo.74.9.561 PMC1042210

[12] Del Prete A Sepe G Ferrante M. Fungal keratitis due to *Scopulariopsis brevicaulis* in an eye previously suffering from herpetic keratitis. Ophthalmologica 1994;208:333–5. 7845651 10.1159/000310533

[13] Ansari Z Miller D Galor A. Current thoughts in fungal keratitis: diagnosis and treatment. Curr Fungal Infect Rep 2013;7:209–18. 24040467 10.1007/s12281-013-0150-110.1007/s12281-013-0150-1 PMC3768010

[14] Matoba AY. Fungal keratitis responsive to moxifloxacin monotherapy. Cornea 2012;31:1206–9. 22673848 10.1097/ICO.0b013e31823f766c PMC3436968

[15] Chodosh J Miller D Tu EY Culbertson WW. Tobramycin-responsive *Fusarium oxysporum* keratitis. Can J Ophthalmol 2000;35:29–30. 10711382 10.1016/s0008-4182(00)80107-6

[16] Khor WB Aung T Saw SM. An outbreak of *Fusarium* keratitis associated with contact lens wear in Singapore. JAMA 2006;295:2867–73. 16804153 10.1001/jama.295.24.2867

[17] Munir WM Rosenfeld SI Udell I. Clinical response of contact lens-associated fungal keratitis to topical fluoroquinolone therapy. Cornea 2007;26:621–4. 17525664 10.1097/ICO.0b013e318033e7e1

[18] Rao SK Lam PT Li EY. A case series of contact lens-associated *Fusarium* keratitis in Hong Kong. Cornea 2007;26:1205–9. 18043177 10.1097/ICO.0b013e31813e32a6

[19] Forster RK Rebell G Wilson LA. Dematiaceous fungal keratitis. Clinical isolates and management. Br J Ophthalmol 1975;59:372–6. 1081406 10.1136/bjo.59.7.372 PMC1042642

[20] Choy BN Tang EW Lai JS. A case series of contact lens associated *Fusarium* keratitis in Hong Kong. Cornea 2009;28:955. 19654511 10.1097/ICO.0b013e3181909351

[21] Lee DC Lee JW Chang SD. A case of *Fusarium* keratitis treated with moxifloxacin 0.5% ophthalmic solution. J Korean Ophthalmol Soc 2012;53:338–41.

[22] Malecha MA. Fungal keratitis caused by *Scopulariopsis brevicaulis* treated successfully with natamycin. Cornea 2004;23:201–3. 15075891 10.1097/00003226-200403000-00015

[23] Donnelly JP Chen SC Kauffman CA. Revision and update of the consensus definitions of invasive fungal disease from the European Organization for Research and Treatment of Cancer and the Mycoses Study Group Education and Research Consortium. Clin Infect Dis 2020;71:1367–76. 31802125 10.1093/cid/ciz1008 PMC7486838

[24] Makimura K Tamura Y Mochizuki T. Phylogenetic classification and species identification of dermatophyte strains based on DNA sequences of nuclear ribosomal internal transcribed spacer 1 regions. J Clin Microbiol 1999;37:920–4. 10074502 10.1128/jcm.37.4.920-924.1999 PMC88625

[25] Dahlgren MA Lingappan A Wilhelmus KR. The clinical diagnosis of microbial keratitis. Am J Ophthalmol 2007;143:940–4. 17408586 10.1016/j.ajo.2007.02.030 PMC1973090

[26] Nixon HK. Preparation of fortified antimicrobial eye drops. Kerala J Ophthalmol 2018;30:152–4.

[27] Zrenner E Tomaszewski K Hamlin J. Effects of multiple doses of voriconazole on the vision of healthy volunteers: a double-blind, placebo-controlled study. Ophthalmic Res 2014;52:43–52. 24925440 10.1159/000359952

[28] Prajna NV Krishnan T Mascarenhas J. The mycotic ulcer treatment trial: a randomized trial comparing natamycin vs voriconazole. JAMA Ophthalmol 2013;131:422–9. 23710492 10.1001/jamaophthalmol.2013.1497 PMC3769211

[29] Gokhale NS. Medical management approach to infectious keratitis. Indian J Ophthalmol 2008;56:215–20. 18417822 10.4103/0301-4738.40360 PMC2636122

[30] Bonifaz A Cruz-Aguilar P Ponce RM. Onychomycosis by molds. Report of 78 cases. Eur J Dermatol 2007;17:70–2. 17324832 10.1684/ejd.2007.0092

[31] Skóra M Bulanda M Jagielski T. In vitro activities of a wide panel of antifungal drugs against various *Scopulariopsis* and *Microascus* species. Antimicrob Agents Chemother 2015;59:5827–9. 26100698 10.1128/AAC.00978-15 PMC4538558

[32] Wang JY Wang DQ Qi XL. Modified ulcer debridement in the treatment of the superficial fungal infection of the cornea. Int J Ophthalmol 2018;11:223–9. 29487810 10.18240/ijo.2018.02.07 PMC5824075

[33] Day S Lalitha P Haug S. Activity of antibiotics against *Fusarium* and *Aspergillus*. Br J Ophthalmol 2009;93:116–9. 18952649 10.1136/bjo.2008.142364 PMC2606932

[34] Lee HB Kim Y Kim JC. Activity of some aminoglycoside antibiotics against true fungi, *Phytophthora* and *Pythium* species. J Appl Microbiol 2005;99:836–43. 16162234 10.1111/j.1365-2672.2005.02684.x

[35] Subedi YP AlFindee MN Takemoto JY. Antifungal amphiphilic kanamycins: new life for an old drug. Med Chem Commun 2018;9:909–19. 10.1039/c8md00155c PMC6071784 30108980

[36] Changa CWT Takemoto JY. Antifungal amphiphilic aminoglycosides. Med Chem Commun 2014;8:1048–57. 10.1039/C4MD00078A PMC4125127 25110571

[37] Alfonso E Miller D. Impact of the 4th generation fluoroquinolones on growth rate and detection time of fungal pathogens. Invest Ophthalmol Vis Sci 2005;46:2766.16043849

[38] Ozdek SC Miller D Flynn PM. In vitro antifungal activity of the fourth generation fluoroquinolones against *Candida* isolates from human ocular infections. Ocul Immunol Inflamm 2006;14:347–51. 17162605 10.1080/09273940600976953

[39] Kawakami H Inuzuka H Hori N. Inhibitory effects of antimicrobial agents against *Fusarium* species. Med Myco 2015;53:603–11. 10.1093/mmy/myv016 25841054

